# Bis{2-[(diisopropyl­phosphan­yl)amino]­pyridine-κ^2^ *N* ^1^,*P*}copper(I) hexa­fluorido­phosphate

**DOI:** 10.1107/S1600536810020283

**Published:** 2010-06-05

**Authors:** Özgür Öztopcu, Karl Kirchner, Kurt Mereiter

**Affiliations:** a Institute of Applied Synthetic Chemistry, Vienna University of Technology, Getreidemarkt 9/163, A-1060 Vienna, Austria; b Institute of Chemical Technologies and Analytics, Vienna University of Technology, Getreidemarkt 9/164SC, A-1060 Vienna, Austria

## Abstract

The crystal structure of the title compound, [Cu(C_11_H_19_N_2_P)_2_]PF_6_, is composed of discrete [Cu(*PN-iPr*)_2_]^+^ cations [*PN-iPr* is 2-(diisopropyl­phosphanyl­amino)­pyridine] and PF_6_^−^ anions. The Cu(I) atom is bis-chelated by two independent *PN-iPr* ligands. It has a distorted tetra­hedral coordination by two P atoms [Cu—P = 2.2277 (4) and 2.2257 (4) Å] and two pyridine N atoms [Cu—N = 2.0763 (11) and 2.0845 (12) Å]. Bond angles about Cu vary from 85.11 (3) (P—Cu—N) to 130.37 (2)° (P—Cu—P). In the crystal, N—H⋯F hydrogen bonds link the Cu complexes and the PF_6_^−^ anions into continuous chains, which show a cross-bedded spatial arrangement. In addition, several weaker C—H⋯F inter­actions contribute to the coherence of the structure.

## Related literature

For the synthesis and crystal structures of *PN*-complexes [*PN* are 2-(phosphanyl­amino)­pyridines], see: Aucott *et al.* (2000 ▶); Benito-Garagorri, Mereiter & Kirchner (2007 ▶); Standfest-Hauser *et al.* (2009 ▶). For applications of *PN*-complexes in catalysis, see: Aguirre *et al.* (2007 ▶); Benito-Garagorri, Wieder­mann *et al.* (2007 ▶). For the chemistry and crystal structures of related *PNP*-complexes [*PNP* = 2,6-bis­(phos­phanyl­amino)­pyridine], see: Benito-Garagorri *et al.* (2006 ▶). For crystal structures of other related Cu(I) complexes, see: Hursthouse *et al.* (2003 ▶); Healy (2008 ▶).
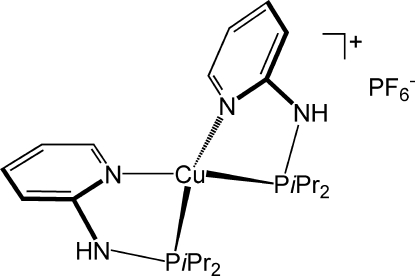


## Experimental

### 

#### Crystal data

[Cu(C_11_H_19_N_2_P)_2_]PF_6_
*M* *_r_* = 629.01
Monoclinic, 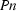

*a* = 11.0357 (13) Å
*b* = 9.2129 (11) Å
*c* = 14.4282 (17) Å
β = 96.723 (1)°
*V* = 1456.8 (3) Å^3^
*Z* = 2
Mo *K*α radiation
μ = 0.97 mm^−1^
*T* = 100 K
0.45 × 0.22 × 0.20 mm

#### Data collection

Bruker APEXII CCD diffractometer
Absorption correction: multi-scan (*SADABS*; Bruker, 2008 ▶) *T*_min_ = 0.65, *T*_max_ = 0.75
21170 measured reflections
8422 independent reflections
8187 reflections with *I* > 2σ(*I*)
*R*_int_ = 0.021

#### Refinement

*R*[*F* ^2^ > 2σ(*F* ^2^)] = 0.021
*wR*(*F* ^2^) = 0.054
*S* = 1.02
8422 reflections
334 parameters
2 restraints
H-atom parameters constrained
Δρ_max_ = 0.35 e Å^−3^
Δρ_min_ = −0.26 e Å^−3^
Absolute structure: Flack (1983 ▶), 4180 Friedel pairs, merohedral twin with twin proportions refined
Flack parameter: 0.410 (4)

### 

Data collection: *APEX2* (Bruker, 2008 ▶); cell refinement: *SAINT* (Bruker, 2008 ▶); data reduction: *SAINT*, *SADABS* and *XPREP* (Bruker, 2008 ▶); program(s) used to solve structure: *SHELXS97* (Sheldrick, 2008 ▶); program(s) used to refine structure: *SHELXL97* (Sheldrick, 2008 ▶); molecular graphics: *Mercury* (Macrae *et al.*, 2006 ▶); software used to prepare material for publication: *publCIF* (Westrip, 2010 ▶).

## Supplementary Material

Crystal structure: contains datablocks global, I. DOI: 10.1107/S1600536810020283/zs2038sup1.cif

Structure factors: contains datablocks I. DOI: 10.1107/S1600536810020283/zs2038Isup2.hkl

Additional supplementary materials:  crystallographic information; 3D view; checkCIF report

## Figures and Tables

**Table 1:** Hydrogen-bond geometry (Å, °)

*D*—H⋯*A*	*D*—H	H⋯*A*	*D*⋯*A*	*D*—H⋯*A*
N2—H2*N*⋯F1^i^	0.88	2.24	3.1038 (15)	167
N4—H4*N*⋯F3^ii^	0.88	2.26	3.1185 (16)	167
C2—H2⋯F4^i^	0.95	2.41	3.306 (2)	158
C20—H20⋯F4	1.00	2.53	3.502 (2)	164

Symmetry codes: (i) 
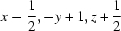
; (ii) 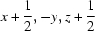.

**Fig. 1.**
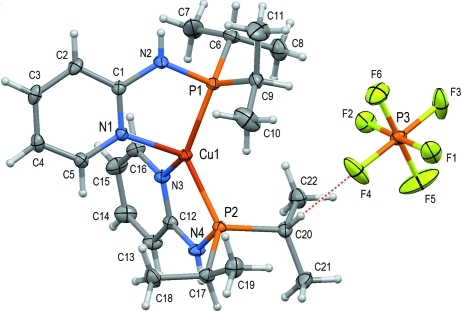
The asymmetric unit of (I) with displacement ellipsoids for the non-hydrogen atoms drawn at the 50% probability level. The red broken line shows one of the C—H···F interactions listed in Table 1.

**Fig. 2.**
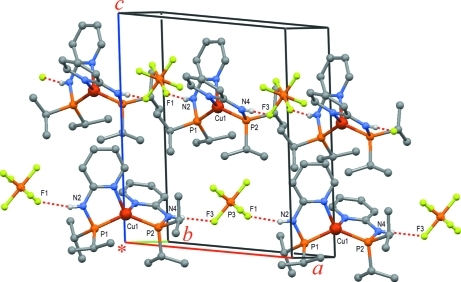
Packing diagram of (I) showing two N—H···F hydrogen bonded chains of [Cu(PN-iPr)2]+ cations and PF6- anions. Upper chain extends parallel to [110], lower chain parallel to [110], C-bonded H atoms omitted for clarity.

## supplementary crystallographic information

### Comment

Heterobidentate pyridylphosphane ligands (*PN*-ligands) in which 2-pyridyl and phosphane moieties are linked by an amino group as spacer are of interest in organometallic chemistry because they contain both hard (nitrogen) and soft (phosphorus) donor atoms at P—N distances of about 3 Å, very suitable for the chelation of transition metals. Moreover, they are readily accessible in a modular fashion *via* the ease of phosphane-nitrogen bond-forming reactions (e.g. Benito-Garagorri *et al.*, 2006). Some transition metal complexes of these ligands, mainly with diphenylphosphane moieties, are catalytically active and have been applied for carbonylation of olefins and other transformations (Aguirre *et al.*, 2007; Benito-Garagorri, Wiedermann *et al.*, 2007). In continuation of earlier work on Ni(II), Pd(II), and Mo(0/II)-complexes of such ligands (Benito-Garagorri, Mereiter & Kirchner, 2007; Standfest-Hauser *et al.*, 2009) we recently focused on Cu(I) complexes and report here the synthesis and crystal structure of the title compound [Cu(*PN-iPr*)_2_]PF_6_. A view of the asymmetric unit is shown in Fig. 1. Copper is bis-chelated by two independent *PN-iPr* ligands and adopts a strongly distorted tetrahedral coordination by two P and two N atoms with well-balanced bond distances of Cu1—P1 = 2.2277 (4) Å, Cu1—P2 = 2.2257 (4) Å, Cu1—N1 = 2.0763 (11) Å, and Cu1—N3 = 2.0845 (12) Å. Bond angles about Cu vary from *ca.* 85° for the two bite angles P1—Cu1—N1 and P2—Cu1—N3 to 130.37 (2)° for P1—Cu1—P2. The twist angle between the planes P1—Cu1—P2 and N1—Cu—N3 is 60.50 (3)° and thus about 30° off from 90°, the value for an ideal undistorted coordination tetrahedron. The complex approaches a molecular non-crystallographic *C*_2_ symmetry, which makes both *PN-iPr* ligands pseudo-equivalent; this includes also the isopropyl groups and their orientations (Fig. 1). A Cu(I)(*PN*)_2_ complex closely related in ligand characteristics to that of the title compound was reported for bis(6-chloro-2-(diphenylphosphinoamino)benzothiazole)copper(I) hexafluoridophosphate (Hursthouse *et al.*, 2003); it has Cu—P ≈ 2.26 Å, Cu—N ≈ 2.06 Å, and a twist angle P—Cu—P *vs.* N—Cu—N of 63.2°. Cu(I) complexes with separate non-chelating two P- and two N-ligands have generally more regular CuP_2_N_2_ tetrahedra with twist angles P—Cu—P *vs.* N—Cu—N near 90°, e.g. bis(pyridine)-bis(triphenylphosphane)copper(I) tetrafluoridoborate (Healy, 2008).

A characteristic feature of *PN-iPr* ligands and their homologues is the acidity of the N—H bond (here N2—H2*N* and N4—H4*N*), which is a good hydrogen bond donor. In the title compound each NH group is hydrogen bonded to the F-atom of an adjacent PF_6_ octahedron at N···F distances of *ca.* 3.1 Å (Table 1). These hydrogen bonds link the cation and anion complexes into infinite chains, which extend parallel to [110] at z ≈ 0.15 and parallel to [110] at z ≈ 0.65 resulting in a cross-bedded arangement (Fig. 2). Several weaker C—H···F interactions contribute to the coherence of the structure. The most significant two of them with H···F < 2.6 Å are included in Table 1, seven more have H···F distances in the range 2.60 to 2.70 Å.

### Experimental

To a solution of [Cu(CH_3_CN)_4_]PF_6_ (100 mg, 0.27 mmol) in THF (10 ml) 2-(diisopropylphosphanylamino)pyridine (*PN-iPr*; 112.8 mg, 0.54 mmol; for synthesis, see: Benito-Garagorri, Mereiter & Kirchner, 2007) was added and the solution was stirred for 12 h. After removal of the solvent a white powder was obtained which was washed with Et_2_O and dried under vacuum. Yield: 100 mg (89%). ^1^H-NMR (δ, acetone, 20 °C): 7.83 (s, 1H, py), 7.69 (s, 1H, py), 7.23 (s, 1H, py), 7.06 (s, 1H, py), 6.78 (s, 1H, NH), 2.90 (s, 2H, CH), 1.22 (s, 12H, CH_3_). ^31^P{^1^H} NMR (δ, acetone, 20 °C): 50.53. Colourless crystals for X-ray diffraction were obtained by evaporation crystallization from acetone.

### Refinement

All H atoms were placed in calculated positions and thereafter treated as riding. A torsional parameter was refined for each methyl group. *U*_iso_(H) = 1.2*U*_eq_(C_non-methyl_) and *U*_iso_(H) = 1.5*U*_eq_(C_methyl_) were used. The title compound is racemic but crystallizes in a non-centrosymmetric polar lattice, space group *P*n. The Flack test indicated that the investigated crystal is a merohedral (polar) twin. In the final refinement this was taken into account by the use of the instructions TWIN and BASF of program *SHELXL97* (Sheldrick, 2008). According to this refinement the amounts of the two twin components are 0.590 (4) and 0.410 (4).

### Crystal data

**Table:**

[Cu(C_11_H_19_N_2_P)_2_]PF_6_	*F*(000) = 652
*M**_r_* = 629.01	*D*_x_ = 1.434 Mg m^−^^3^
Monoclinic, *P**n*	Mo *K*α radiation, λ = 0.71073 Å
Hall symbol: P -2yac	Cell parameters from 9966 reflections
*a* = 11.0357 (13) Å	θ = 2.5–30.0°
*b* = 9.2129 (11) Å	µ = 0.97 mm^−^^1^
*c* = 14.4282 (17) Å	*T* = 100 K
β = 96.723 (1)°	Block, colourless
*V* = 1456.8 (3) Å^3^	0.45 × 0.22 × 0.20 mm
*Z* = 2	

### Data collection

**Table:**

Bruker APEXII CCD diffractometer	8422 independent reflections
Radiation source: fine-focus sealed tube	8187 reflections with *I* > 2σ(*I*)
graphite	*R*_int_ = 0.021
φ and ω scans	θ_max_ = 30.0°, θ_min_ = 2.6°
Absorption correction: multi-scan (*SADABS*; Bruker, 2008)	*h* = −15→15
*T*_min_ = 0.65, *T*_max_ = 0.75	*k* = −12→12
21170 measured reflections	*l* = −20→20

### Refinement

**Table:**

Refinement on *F*^2^	Secondary atom site location: difference Fourier map
Least-squares matrix: full	Hydrogen site location: inferred from neighbouring sites
*R*[*F*^2^ > 2σ(*F*^2^)] = 0.021	H-atom parameters constrained
*wR*(*F*^2^) = 0.054	*w* = 1/[σ^2^(*F*_o_^2^) + (0.0327*P*)^2^ + 0.1922*P*] where *P* = (*F*_o_^2^ + 2*F*_c_^2^)/3
*S* = 1.02	(Δ/σ)_max_ = 0.001
8422 reflections	Δρ_max_ = 0.35 e Å^−^^3^
334 parameters	Δρ_min_ = −0.26 e Å^−^^3^
2 restraints	Absolute structure: Flack (1983), 4180 Friedel pairs, merohedral twin with twin proportions refined
Primary atom site location: structure-invariant direct methods	Flack parameter: 0.410 (4)

### Special details

**Table:**

Geometry. All esds (except the esd in the dihedral angle between two l.s. planes) are estimated using the full covariance matrix. The cell esds are taken into account individually in the estimation of esds in distances, angles and torsion angles; correlations between esds in cell parameters are only used when they are defined by crystal symmetry. An approximate (isotropic) treatment of cell esds is used for estimating esds involving l.s. planes.
Refinement. Refinement of *F*^2^ against ALL reflections. The weighted *R*-factor wR and goodness of fit *S* are based on *F*^2^, conventional *R*-factors *R* are based on *F*, with *F* set to zero for negative *F*^2^. The threshold expression of *F*^2^ > σ(*F*^2^) is used only for calculating *R*-factors(gt) etc. and is not relevant to the choice of reflections for refinement. *R*-factors based on *F*^2^ are statistically about twice as large as those based on *F*, and *R*- factors based on ALL data will be even larger.

### Fractional atomic coordinates and isotropic or equivalent isotropic displacement parameters (Å^2^)

**Table:**

	*x*	*y*	*z*	*U*_iso_*/*U*_eq_	
Cu1	0.545130 (11)	0.141471 (15)	0.626623 (10)	0.01477 (4)	
P1	0.38719 (3)	0.26283 (4)	0.55383 (2)	0.01685 (6)	
P2	0.72626 (3)	0.09024 (3)	0.58123 (2)	0.01465 (6)	
N1	0.50957 (10)	0.25486 (12)	0.74483 (8)	0.0155 (2)	
N2	0.34515 (12)	0.35253 (13)	0.64884 (8)	0.0209 (2)	
H2N	0.2830	0.4129	0.6411	0.025*	
N3	0.54844 (10)	−0.07232 (12)	0.67416 (8)	0.0168 (2)	
N4	0.74461 (10)	−0.07879 (12)	0.62954 (8)	0.0180 (2)	
H4N	0.8145	−0.1241	0.6281	0.022*	
C1	0.40583 (12)	0.33308 (14)	0.73755 (9)	0.0164 (2)	
C2	0.36015 (13)	0.39407 (15)	0.81595 (10)	0.0198 (2)	
H2	0.2852	0.4459	0.8095	0.024*	
C3	0.42655 (14)	0.37681 (15)	0.90213 (10)	0.0215 (3)	
H3	0.3978	0.4173	0.9560	0.026*	
C4	0.53606 (13)	0.29988 (15)	0.91019 (9)	0.0207 (3)	
H4	0.5842	0.2893	0.9688	0.025*	
C5	0.57229 (12)	0.23970 (14)	0.83056 (9)	0.0178 (2)	
H5	0.6455	0.1844	0.8362	0.021*	
C6	0.24294 (13)	0.18007 (18)	0.50231 (11)	0.0249 (3)	
H6	0.1814	0.2584	0.4860	0.030*	
C7	0.26381 (16)	0.0977 (2)	0.41411 (12)	0.0325 (3)	
H7A	0.3187	0.0157	0.4304	0.049*	
H7B	0.3005	0.1629	0.3715	0.049*	
H7C	0.1856	0.0616	0.3836	0.049*	
C8	0.19540 (16)	0.0774 (3)	0.57232 (13)	0.0435 (5)	
H8A	0.2568	0.0027	0.5906	0.065*	
H8B	0.1201	0.0311	0.5439	0.065*	
H8C	0.1786	0.1321	0.6276	0.065*	
C9	0.41591 (14)	0.40856 (16)	0.47148 (10)	0.0228 (3)	
H9	0.4234	0.3609	0.4101	0.027*	
C10	0.53711 (19)	0.4818 (2)	0.50186 (15)	0.0423 (4)	
H10A	0.5560	0.5500	0.4535	0.063*	
H10B	0.6015	0.4082	0.5114	0.063*	
H10C	0.5322	0.5344	0.5603	0.063*	
C11	0.3130 (2)	0.5190 (3)	0.45456 (15)	0.0473 (5)	
H11A	0.3089	0.5764	0.5113	0.071*	
H11B	0.2354	0.4682	0.4383	0.071*	
H11C	0.3285	0.5835	0.4032	0.071*	
C12	0.65306 (12)	−0.14630 (13)	0.67082 (9)	0.0161 (2)	
C13	0.67045 (13)	−0.28684 (15)	0.70895 (11)	0.0218 (3)	
H13	0.7447	−0.3376	0.7055	0.026*	
C14	0.57773 (14)	−0.34880 (15)	0.75124 (12)	0.0269 (3)	
H14	0.5874	−0.4432	0.7775	0.032*	
C15	0.46883 (14)	−0.27208 (17)	0.75539 (12)	0.0280 (3)	
H15	0.4039	−0.3128	0.7846	0.034*	
C16	0.45880 (13)	−0.13615 (15)	0.71590 (11)	0.0220 (3)	
H16	0.3848	−0.0843	0.7181	0.026*	
C17	0.87311 (12)	0.17551 (15)	0.62197 (10)	0.0195 (2)	
H17	0.9400	0.1074	0.6099	0.023*	
C18	0.88851 (16)	0.31683 (17)	0.56853 (12)	0.0278 (3)	
H18A	0.8217	0.3834	0.5778	0.042*	
H18B	0.8871	0.2955	0.5019	0.042*	
H18C	0.9666	0.3620	0.5918	0.042*	
C19	0.88143 (15)	0.20342 (19)	0.72714 (10)	0.0278 (3)	
H19A	0.9629	0.2401	0.7497	0.042*	
H19B	0.8667	0.1126	0.7594	0.042*	
H19C	0.8200	0.2754	0.7396	0.042*	
C20	0.73108 (13)	0.05665 (15)	0.45568 (9)	0.0205 (2)	
H20	0.7185	0.1517	0.4224	0.025*	
C21	0.62513 (16)	−0.0434 (2)	0.41957 (11)	0.0319 (3)	
H21A	0.6225	−0.0543	0.3518	0.048*	
H21B	0.5482	−0.0012	0.4344	0.048*	
H21C	0.6369	−0.1387	0.4494	0.048*	
C22	0.85233 (15)	−0.00667 (18)	0.43271 (11)	0.0284 (3)	
H22A	0.8741	−0.0907	0.4729	0.043*	
H22B	0.9163	0.0672	0.4434	0.043*	
H22C	0.8441	−0.0371	0.3672	0.043*	
P3	0.55962 (3)	0.31567 (4)	0.21299 (3)	0.01976 (7)	
F1	0.62918 (10)	0.43352 (14)	0.15595 (8)	0.0398 (3)	
F2	0.48767 (9)	0.20025 (11)	0.26926 (7)	0.0333 (2)	
F3	0.46624 (12)	0.28652 (18)	0.12151 (7)	0.0542 (4)	
F4	0.65102 (12)	0.34760 (14)	0.30470 (9)	0.0516 (4)	
F5	0.64776 (15)	0.19392 (16)	0.18453 (16)	0.0785 (6)	
F6	0.47085 (12)	0.43906 (12)	0.24247 (9)	0.0434 (3)	

### Atomic displacement parameters (Å^2^)

**Table:**

	*U*^11^	*U*^22^	*U*^33^	*U*^12^	*U*^13^	*U*^23^
Cu1	0.01443 (6)	0.01369 (7)	0.01602 (7)	0.00390 (6)	0.00110 (5)	−0.00054 (6)
P1	0.01635 (13)	0.01949 (15)	0.01425 (13)	0.00588 (12)	−0.00017 (11)	−0.00045 (12)
P2	0.01461 (13)	0.01342 (14)	0.01588 (14)	0.00242 (11)	0.00163 (10)	0.00086 (11)
N1	0.0170 (5)	0.0150 (5)	0.0149 (5)	0.0012 (4)	0.0026 (4)	0.0010 (4)
N2	0.0227 (5)	0.0232 (6)	0.0166 (5)	0.0126 (4)	0.0011 (4)	0.0002 (4)
N3	0.0160 (5)	0.0143 (5)	0.0201 (5)	0.0010 (4)	0.0017 (4)	−0.0007 (4)
N4	0.0152 (5)	0.0154 (5)	0.0236 (5)	0.0045 (4)	0.0031 (4)	0.0051 (4)
C1	0.0191 (6)	0.0135 (5)	0.0166 (6)	0.0022 (4)	0.0026 (4)	0.0003 (4)
C2	0.0250 (6)	0.0142 (6)	0.0215 (6)	0.0028 (5)	0.0086 (5)	−0.0007 (5)
C3	0.0300 (7)	0.0174 (6)	0.0184 (6)	−0.0044 (5)	0.0088 (5)	−0.0032 (5)
C4	0.0262 (6)	0.0211 (6)	0.0148 (5)	−0.0068 (5)	0.0019 (5)	0.0010 (5)
C5	0.0181 (5)	0.0168 (6)	0.0181 (6)	−0.0018 (4)	0.0009 (4)	0.0024 (4)
C6	0.0180 (6)	0.0313 (7)	0.0239 (7)	0.0052 (5)	−0.0038 (5)	−0.0009 (6)
C7	0.0363 (8)	0.0344 (8)	0.0256 (7)	−0.0071 (7)	−0.0008 (6)	−0.0060 (6)
C8	0.0217 (7)	0.0752 (15)	0.0336 (9)	−0.0164 (8)	0.0036 (6)	0.0046 (9)
C9	0.0270 (7)	0.0248 (7)	0.0162 (6)	0.0058 (5)	0.0010 (5)	0.0019 (5)
C10	0.0443 (10)	0.0269 (8)	0.0513 (11)	−0.0091 (7)	−0.0123 (8)	0.0144 (8)
C11	0.0472 (11)	0.0484 (12)	0.0475 (11)	0.0239 (9)	0.0106 (9)	0.0275 (9)
C12	0.0150 (5)	0.0146 (5)	0.0182 (6)	0.0005 (4)	0.0000 (4)	−0.0010 (4)
C13	0.0204 (6)	0.0139 (6)	0.0304 (7)	0.0019 (5)	0.0007 (5)	0.0021 (5)
C14	0.0256 (7)	0.0151 (6)	0.0399 (9)	−0.0017 (5)	0.0038 (6)	0.0056 (6)
C15	0.0242 (7)	0.0209 (7)	0.0406 (8)	−0.0029 (5)	0.0105 (6)	0.0057 (6)
C16	0.0175 (6)	0.0200 (6)	0.0290 (7)	0.0006 (5)	0.0056 (5)	0.0007 (5)
C17	0.0178 (6)	0.0192 (6)	0.0216 (6)	−0.0013 (5)	0.0020 (5)	0.0007 (5)
C18	0.0316 (7)	0.0235 (7)	0.0287 (7)	−0.0067 (6)	0.0056 (6)	0.0017 (6)
C19	0.0268 (7)	0.0336 (8)	0.0217 (7)	−0.0065 (6)	−0.0034 (5)	−0.0007 (6)
C20	0.0250 (6)	0.0197 (6)	0.0169 (6)	0.0061 (5)	0.0024 (5)	0.0005 (5)
C21	0.0324 (8)	0.0368 (9)	0.0251 (7)	0.0005 (7)	−0.0024 (6)	−0.0088 (6)
C22	0.0309 (7)	0.0307 (8)	0.0253 (6)	0.0071 (6)	0.0104 (6)	−0.0018 (6)
P3	0.01635 (14)	0.02039 (15)	0.02286 (16)	−0.00373 (12)	0.00365 (12)	−0.00201 (13)
F1	0.0306 (5)	0.0534 (7)	0.0353 (5)	−0.0215 (5)	0.0030 (4)	0.0106 (5)
F2	0.0329 (5)	0.0303 (5)	0.0366 (5)	−0.0113 (4)	0.0039 (4)	0.0085 (4)
F3	0.0530 (7)	0.0899 (10)	0.0193 (4)	−0.0476 (7)	0.0026 (4)	−0.0051 (5)
F4	0.0451 (7)	0.0571 (8)	0.0453 (7)	−0.0261 (6)	−0.0252 (5)	0.0224 (6)
F5	0.0594 (9)	0.0427 (8)	0.1435 (17)	0.0108 (7)	0.0543 (10)	−0.0213 (9)
F6	0.0505 (6)	0.0329 (6)	0.0500 (6)	0.0158 (5)	0.0188 (5)	0.0059 (5)

### Geometric parameters (Å, °)

**Table:**

Cu1—N1	2.0763 (11)	C10—H10A	0.9800
Cu1—N3	2.0845 (12)	C10—H10B	0.9800
Cu1—P2	2.2257 (4)	C10—H10C	0.9800
Cu1—P1	2.2277 (4)	C11—H11A	0.9800
P1—N2	1.7107 (12)	C11—H11B	0.9800
P1—C6	1.8415 (16)	C11—H11C	0.9800
P1—C9	1.8446 (15)	C12—C13	1.4112 (18)
P2—N4	1.7084 (12)	C13—C14	1.375 (2)
P2—C17	1.8347 (14)	C13—H13	0.9500
P2—C20	1.8446 (14)	C14—C15	1.401 (2)
N1—C1	1.3464 (16)	C14—H14	0.9500
N1—C5	1.3521 (16)	C15—C16	1.375 (2)
N2—C1	1.3850 (17)	C15—H15	0.9500
N2—H2N	0.8800	C16—H16	0.9500
N3—C12	1.3464 (16)	C17—C19	1.531 (2)
N3—C16	1.3516 (18)	C17—C18	1.533 (2)
N4—C12	1.3795 (17)	C17—H17	1.0000
N4—H4N	0.8800	C18—H18A	0.9800
C1—C2	1.4080 (18)	C18—H18B	0.9800
C2—C3	1.377 (2)	C18—H18C	0.9800
C2—H2	0.9500	C19—H19A	0.9800
C3—C4	1.394 (2)	C19—H19B	0.9800
C3—H3	0.9500	C19—H19C	0.9800
C4—C5	1.3766 (19)	C20—C22	1.531 (2)
C4—H4	0.9500	C20—C21	1.531 (2)
C5—H5	0.9500	C20—H20	1.0000
C6—C8	1.522 (3)	C21—H21A	0.9800
C6—C7	1.522 (2)	C21—H21B	0.9800
C6—H6	1.0000	C21—H21C	0.9800
C7—H7A	0.9800	C22—H22A	0.9800
C7—H7B	0.9800	C22—H22B	0.9800
C7—H7C	0.9800	C22—H22C	0.9800
C8—H8A	0.9800	P3—F5	1.5704 (13)
C8—H8B	0.9800	P3—F6	1.5907 (11)
C8—H8C	0.9800	P3—F4	1.5944 (12)
C9—C10	1.516 (2)	P3—F3	1.5993 (11)
C9—C11	1.523 (2)	P3—F2	1.6036 (10)
C9—H9	1.0000	P3—F1	1.6103 (11)
			
N1—Cu1—N3	101.70 (4)	H10B—C10—H10C	109.5
N1—Cu1—P2	127.66 (3)	C9—C11—H11A	109.5
N3—Cu1—P2	85.11 (3)	C9—C11—H11B	109.5
N1—Cu1—P1	85.53 (3)	H11A—C11—H11B	109.5
N3—Cu1—P1	127.73 (3)	C9—C11—H11C	109.5
P2—Cu1—P1	130.374 (15)	H11A—C11—H11C	109.5
N2—P1—C6	102.75 (7)	H11B—C11—H11C	109.5
N2—P1—C9	104.34 (7)	N3—C12—N4	117.49 (12)
C6—P1—C9	104.30 (7)	N3—C12—C13	121.91 (12)
N2—P1—Cu1	97.78 (4)	N4—C12—C13	120.59 (12)
C6—P1—Cu1	125.04 (5)	C14—C13—C12	118.66 (13)
C9—P1—Cu1	119.02 (5)	C14—C13—H13	120.7
N4—P2—C17	101.62 (6)	C12—C13—H13	120.7
N4—P2—C20	103.42 (6)	C13—C14—C15	119.73 (13)
C17—P2—C20	105.10 (6)	C13—C14—H14	120.1
N4—P2—Cu1	98.10 (4)	C15—C14—H14	120.1
C17—P2—Cu1	127.23 (5)	C16—C15—C14	118.00 (13)
C20—P2—Cu1	117.04 (5)	C16—C15—H15	121.0
C1—N1—C5	117.79 (11)	C14—C15—H15	121.0
C1—N1—Cu1	116.49 (9)	N3—C16—C15	123.59 (13)
C5—N1—Cu1	125.01 (9)	N3—C16—H16	118.2
C1—N2—P1	122.07 (9)	C15—C16—H16	118.2
C1—N2—H2N	119.0	C19—C17—C18	111.02 (13)
P1—N2—H2N	119.0	C19—C17—P2	109.74 (10)
C12—N3—C16	118.11 (12)	C18—C17—P2	110.39 (10)
C12—N3—Cu1	116.65 (9)	C19—C17—H17	108.5
C16—N3—Cu1	124.90 (9)	C18—C17—H17	108.5
C12—N4—P2	121.95 (9)	P2—C17—H17	108.5
C12—N4—H4N	119.0	C17—C18—H18A	109.5
P2—N4—H4N	119.0	C17—C18—H18B	109.5
N1—C1—N2	117.13 (12)	H18A—C18—H18B	109.5
N1—C1—C2	122.14 (12)	C17—C18—H18C	109.5
N2—C1—C2	120.73 (12)	H18A—C18—H18C	109.5
C3—C2—C1	118.48 (13)	H18B—C18—H18C	109.5
C3—C2—H2	120.8	C17—C19—H19A	109.5
C1—C2—H2	120.8	C17—C19—H19B	109.5
C2—C3—C4	119.91 (12)	H19A—C19—H19B	109.5
C2—C3—H3	120.0	C17—C19—H19C	109.5
C4—C3—H3	120.0	H19A—C19—H19C	109.5
C5—C4—C3	117.96 (13)	H19B—C19—H19C	109.5
C5—C4—H4	121.0	C22—C20—C21	110.42 (13)
C3—C4—H4	121.0	C22—C20—P2	113.76 (10)
N1—C5—C4	123.66 (13)	C21—C20—P2	109.05 (10)
N1—C5—H5	118.2	C22—C20—H20	107.8
C4—C5—H5	118.2	C21—C20—H20	107.8
C8—C6—C7	110.05 (15)	P2—C20—H20	107.8
C8—C6—P1	109.78 (11)	C20—C21—H21A	109.5
C7—C6—P1	109.61 (11)	C20—C21—H21B	109.5
C8—C6—H6	109.1	H21A—C21—H21B	109.5
C7—C6—H6	109.1	C20—C21—H21C	109.5
P1—C6—H6	109.1	H21A—C21—H21C	109.5
C6—C7—H7A	109.5	H21B—C21—H21C	109.5
C6—C7—H7B	109.5	C20—C22—H22A	109.5
H7A—C7—H7B	109.5	C20—C22—H22B	109.5
C6—C7—H7C	109.5	H22A—C22—H22B	109.5
H7A—C7—H7C	109.5	C20—C22—H22C	109.5
H7B—C7—H7C	109.5	H22A—C22—H22C	109.5
C6—C8—H8A	109.5	H22B—C22—H22C	109.5
C6—C8—H8B	109.5	F5—P3—F6	179.62 (11)
H8A—C8—H8B	109.5	F5—P3—F4	89.87 (10)
C6—C8—H8C	109.5	F6—P3—F4	89.76 (8)
H8A—C8—H8C	109.5	F5—P3—F3	91.33 (10)
H8B—C8—H8C	109.5	F6—P3—F3	89.04 (8)
C10—C9—C11	111.42 (16)	F4—P3—F3	178.79 (9)
C10—C9—P1	110.47 (11)	F5—P3—F2	90.99 (8)
C11—C9—P1	114.10 (12)	F6—P3—F2	88.94 (6)
C10—C9—H9	106.8	F4—P3—F2	90.28 (6)
C11—C9—H9	106.8	F3—P3—F2	89.83 (6)
P1—C9—H9	106.8	F5—P3—F1	90.06 (8)
C9—C10—H10A	109.5	F6—P3—F1	90.01 (7)
C9—C10—H10B	109.5	F4—P3—F1	90.38 (6)
H10A—C10—H10B	109.5	F3—P3—F1	89.49 (6)
C9—C10—H10C	109.5	F2—P3—F1	178.76 (6)
H10A—C10—H10C	109.5		
			
N1—Cu1—P1—N2	−3.58 (6)	N2—C1—C2—C3	−177.28 (13)
N3—Cu1—P1—N2	97.77 (6)	C1—C2—C3—C4	−0.2 (2)
P2—Cu1—P1—N2	−141.14 (5)	C2—C3—C4—C5	−1.8 (2)
N1—Cu1—P1—C6	−115.16 (7)	C1—N1—C5—C4	−0.2 (2)
N3—Cu1—P1—C6	−13.82 (8)	Cu1—N1—C5—C4	−170.10 (10)
P2—Cu1—P1—C6	107.27 (6)	C3—C4—C5—N1	2.1 (2)
N1—Cu1—P1—C9	107.62 (6)	N2—P1—C6—C8	−60.40 (14)
N3—Cu1—P1—C9	−151.03 (7)	C9—P1—C6—C8	−169.05 (13)
P2—Cu1—P1—C9	−29.94 (6)	Cu1—P1—C6—C8	48.75 (14)
N1—Cu1—P2—N4	94.55 (6)	N2—P1—C6—C7	178.60 (11)
N3—Cu1—P2—N4	−6.47 (5)	C9—P1—C6—C7	69.95 (13)
P1—Cu1—P2—N4	−143.64 (4)	Cu1—P1—C6—C7	−72.24 (13)
N1—Cu1—P2—C17	−16.78 (7)	N2—P1—C9—C10	75.95 (13)
N3—Cu1—P2—C17	−117.80 (7)	C6—P1—C9—C10	−176.57 (13)
P1—Cu1—P2—C17	105.03 (6)	Cu1—P1—C9—C10	−31.59 (14)
N1—Cu1—P2—C20	−155.83 (6)	N2—P1—C9—C11	−50.50 (15)
N3—Cu1—P2—C20	103.16 (6)	C6—P1—C9—C11	56.98 (15)
P1—Cu1—P2—C20	−34.02 (6)	Cu1—P1—C9—C11	−158.05 (13)
N3—Cu1—N1—C1	−118.85 (9)	C16—N3—C12—N4	−178.83 (12)
P2—Cu1—N1—C1	148.29 (8)	Cu1—N3—C12—N4	−5.15 (16)
P1—Cu1—N1—C1	8.79 (9)	C16—N3—C12—C13	0.3 (2)
N3—Cu1—N1—C5	51.21 (11)	Cu1—N3—C12—C13	173.97 (10)
P2—Cu1—N1—C5	−41.64 (12)	P2—N4—C12—N3	−1.76 (17)
P1—Cu1—N1—C5	178.85 (11)	P2—N4—C12—C13	179.10 (11)
C6—P1—N2—C1	127.56 (12)	N3—C12—C13—C14	−0.5 (2)
C9—P1—N2—C1	−123.82 (12)	N4—C12—C13—C14	178.60 (14)
Cu1—P1—N2—C1	−1.12 (12)	C12—C13—C14—C15	0.1 (2)
N1—Cu1—N3—C12	−120.07 (10)	C13—C14—C15—C16	0.4 (2)
P2—Cu1—N3—C12	7.42 (9)	C12—N3—C16—C15	0.3 (2)
P1—Cu1—N3—C12	146.52 (8)	Cu1—N3—C16—C15	−172.84 (12)
N1—Cu1—N3—C16	53.13 (12)	C14—C15—C16—N3	−0.6 (3)
P2—Cu1—N3—C16	−179.38 (11)	N4—P2—C17—C19	−70.36 (11)
P1—Cu1—N3—C16	−40.29 (13)	C20—P2—C17—C19	−177.88 (10)
C17—P2—N4—C12	137.45 (11)	Cu1—P2—C17—C19	39.33 (12)
C20—P2—N4—C12	−113.74 (11)	N4—P2—C17—C18	166.96 (10)
Cu1—P2—N4—C12	6.67 (11)	C20—P2—C17—C18	59.45 (12)
C5—N1—C1—N2	177.49 (12)	Cu1—P2—C17—C18	−83.34 (11)
Cu1—N1—C1—N2	−11.70 (15)	N4—P2—C20—C22	−62.05 (12)
C5—N1—C1—C2	−2.08 (19)	C17—P2—C20—C22	44.15 (12)
Cu1—N1—C1—C2	168.72 (10)	Cu1—P2—C20—C22	−168.57 (9)
P1—N2—C1—N1	8.39 (18)	N4—P2—C20—C21	61.68 (11)
P1—N2—C1—C2	−172.03 (11)	C17—P2—C20—C21	167.88 (10)
N1—C1—C2—C3	2.3 (2)	Cu1—P2—C20—C21	−44.84 (11)

### Hydrogen-bond geometry (Å, °)

**Table:**

*D*—H···*A*	*D*—H	H···*A*	*D*···*A*	*D*—H···*A*
N2—H2N···F1^i^	0.88	2.24	3.1038 (15)	167
N4—H4N···F3^ii^	0.88	2.26	3.1185 (16)	167
C2—H2···F4^i^	0.95	2.41	3.306 (2)	158
C20—H20···F4	1.00	2.53	3.502 (2)	164

Symmetry codes: (i) *x*−1/2, −*y*+1, *z*+1/2; (ii) *x*+1/2, −*y*, *z*+1/2.
